# An extra-cardiac lesion with pseudo-kidney sign detected by transthoracic echocardiography

**DOI:** 10.1093/ehjcr/ytae311

**Published:** 2024-07-02

**Authors:** Satoshi Kurisu, Hitoshi Fujiwara, Hiroko Todo, Yoshiro Tachiyama

**Affiliations:** Department of Cardiology, NHO Hiroshimanishi Medical Center, 4-1-1, Kuba, 739-0696 Otake, Japan; Department of Cardiology, NHO Hiroshimanishi Medical Center, 4-1-1, Kuba, 739-0696 Otake, Japan; Department of Gastroenterology, NHO Hiroshimanishi Medical Center, Otake, Japan; Department of Diagnostic Pathology, NHO Hiroshimanishi Medical Center, Otake, Japan

## Case description

Transthoracic echocardiography (TTE) is a widely used modality for the assessment of cardiac structure and function. We report a patient with an extra-cardiac lesion detected by TTE, leading to a diagnosis of gastric cancer.

A 79-year-old man with dyspnoea and lower extremity oedema persisting for 2 weeks was referred to our hospital. He presented with weight loss of 4 kg over the past 3 months. The patient had no significant past medical or drug history.

**Figure 1:**
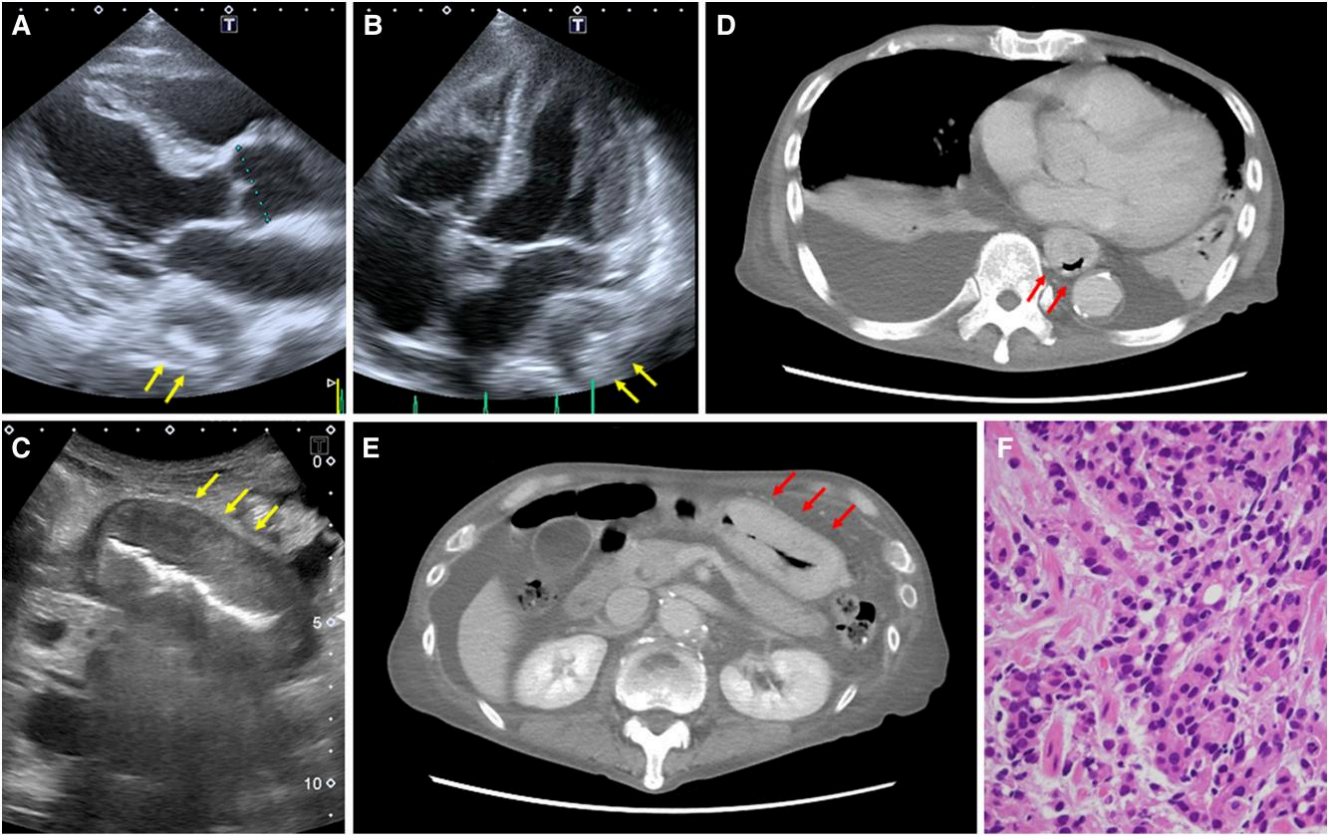
A transthoracic echocardiogram showed an extra-cardiac lesion measuring 33 × 30 mm located immediately adjacent to the left atrium. The lesion was characterized by a large hypoechoic mass with a central hyperechoic area, the so-called pseudo-kidney sign (*A* and *B*, arrows). An abdominal ultrasound also revealed pseudo-kidney sign in the stomach (*C*, arrows). Computed tomography scans showed that these lesions were located in the oesophagus and stomach (*D* and *E*, arrows). The pathological examination showed poorly differentiated gastric adenocarcinoma (*F*).

Laboratory investigations showed haemoglobin of 5.4 g/dL, albumin of 2.2 g/dL, iron of 8 µg/dL, and N-terminal pro-brain natriuretic peptide of 1407 pg/mL. Liver and renal functions were normal. A TTE showed an extra-cardiac lesion measuring 33 × 30 mm located immediately adjacent to the left atrium. The lesion was characterized by a large hypoechoic mass with a central hyperechoic area, the so-called pseudo-kidney sign^1,2^ (*Figure 1A* and *B*, arrows). Left ventricular ejection fraction was 68%, and *E*/*e*′, a surrogate for left ventricular filling pressure, was 10. No significant valvular heart diseases were found. Given these findings, his symptoms were attributed mainly to severe anaemia and hypoalbuminaemia. An abdominal ultrasound also revealed pseudo-kidney sign in the stomach (*Figure 1C*, arrows). Computed tomography scans showed that these lesions were located in the oesophagus and stomach (*Figure 1D* and *E*, arrows). An oesophagogastroscopy was subsequently performed, revealing a wide lesion from the antrum to the cardia, invading the thoracic oesophagus. The pathological examination showed poorly differentiated gastric adenocarcinoma (*Figure 1F*). The patient received palliative care and eventually died on hospital Day 35.

The differential diagnosis of extra-cardiac posterior mediastinal lesions detected by TTE includes metastatic lymph nodes and oesophageal lesions, the majority of which are oesophageal cancers.^3^ Clinicians should be aware that unexpected lesions with pseudo-kidney sign may be detected during routine TTE.

**Consent:** The authors confirm that written consent for submission and publication of this case report including images has been obtained from the patient in line with COPE guidance.

**Funding:** None declared.

## Data Availability

The data that support the findings of this study are available from the corresponding author upon reasonable request.
